# Risk Stratification for Lung Cancer Patients

**DOI:** 10.7759/cureus.30643

**Published:** 2022-10-24

**Authors:** Anchal Jain, Bejoy Philip, Munira Begum, William Wang, Michael Ogunjimi, Amer Harky

**Affiliations:** 1 Cardiothoracic Surgery, Royal Stoke University Hospital, Stoke on Trent, GBR; 2 Thoracic Surgery, Liverpool Heart and Chest Hospital, Liverpool, GBR; 3 Surgery, St George’s Hospital Medical School, St George’s University of London, London, GBR; 4 Surgery, Barts and The London School of Medicine and Dentistry, Queen Mary University of London, London, GBR; 5 Surgery, Imperial College School of Medicine, London, GBR; 6 Cardiothoracic Surgery, Liverpool Heart and Chest Hospital, Liverpool, GBR

**Keywords:** frailty, risk index, exercise test, perioperative evaluation, fitness for surgery, pulmonary function, lung cancer surgery

## Abstract

A comprehensive review of relevant clinical literature on evidence-based recommendations and existing prediction models specific to lung cancer surgery was undertaken. Preoperative risk assessment parameters such as pulmonary function tests (PFT), cardiopulmonary exercise testing (CPET), Brunelli models, Thoracoscore and frailty were analyzed for predicting postoperative risk of complications.

When assessing fitness for surgery, the primarily used PFT parameters such as predictive postoperative forced expiratory volume in one second (FEV1) and diffusion capacity for carbon monoxide* *(DLCO ) showed conflicting evidence in determining a positive correlation with postoperative mortality. CPET variables predicted higher complication risk when VO2peak < 10ml/kg/min, AT < 11ml/kg/min and ventilation/carbon dioxide production (VE/VCO2) was in range of 34-40. While a cardiac risk index like the Thoracic Revised Cardiac Risk Index (ThRCRI) predicted major cardiovascular compromise, a thoracic risk index like Thoracoscore proved imprecise. Lastly, frailty is used to risk stratify patients in clinical practice but a recognized validated model specific to thoracic surgery is non-existent.

When considering patients for lung cancer surgery, some dilemma exists regarding the accuracy of clinical prediction models and their external validation. There is a pressing need for the development of a consolidated clinically robust risk stratification model to predict complications after thoracic resections.

## Introduction and background

Lung cancer is the leading cause of oncological deaths worldwide [1]. It is an invasive and rapidly metastasizing cancer prevalent in people who smoke cigarettes. Smoking causes other lung and vascular disease which increases patient co-morbidities, thereby limiting their ability to tolerate radical treatment. Lung cancer incidence also increases with age which is the biggest risk factor that determines operative mortality and early morbidity. Recent advances in imaging modalities, diagnostic pathways and screening programs, such as the use of low-dose computed tomography in high-risk populations, have allowed for the early detection of lung cancer [1].

Treatment of early-stage lung cancer primarily involves surgical resection as it delivers a strong probability of cure [2]. Despite this, functional impairment as a result of lung resection is a recognized consequence that can cause a series of postoperative complications, including those that are life-threatening. 

This highlights the need to risk stratify to predict mortality, morbidity and long-term shortness of breath in lung cancer patients. In the last decade, an increasing amount of literature has addressed the role of various parameters used in preoperative risk assessment. This often involves cardiorespiratory evaluation in conjunction with assessment of operative mortality, lung function and exercise testing. More recently, there has been an emphasis on frailty as a significant surgical risk factor and practical screening tool. 

Individual preoperative investigations such as pulmonary function tests (PFT), cardiopulmonary exercise testing (CPET), Brunelli models, Thoracoscore and frailty can be useful in clinical practice and the accuracy of these in predicting postoperative risk has been analyzed in this article. 

**Figure 1:**
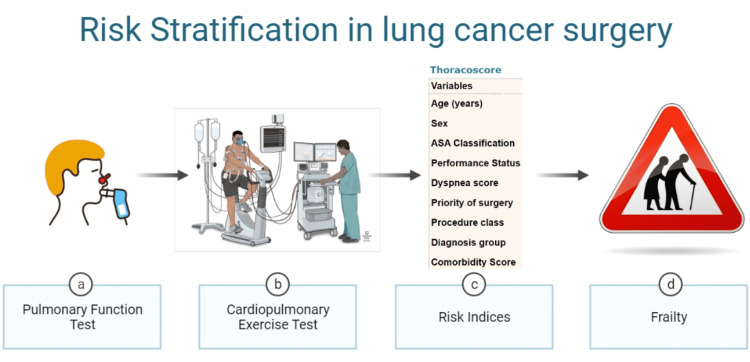
Risk stratification modalities for lung cancer surgery patients Image courtesy of the authors.

## Review

Pulmonary function tests

ppoFEV1

Assessment of pulmonary function parameters such as forced expiratory volume in one second (FEV1) and predicted postoperative FEV1 (ppoFEV1) is important in-patient selection for lung cancer surgery. This is to evaluate the possibility of postoperative pulmonary complications (PPC) and operative mortality, defined as death within 30 days of surgery. Patients with an estimated ppoFEV1>40% are considered as having an average risk, and high risk is determined by an estimated ppoFEV1 <40% [3]. In the last few years, numerous studies have aimed to elucidate the correlation between pulmonary function tests performed before surgery and postoperative risks. 

Various small studies have been carried out at local or regional units to establish the risk indicators for developing PPC. A four-year prospective observational study of 285 patients undergoing video-assisted thoracoscopic surgery (VATS) for non-small cell lung cancer (NSCLC) was carried out. Data on gender, age, predicted %FEV1, ASA score, perioperative activity level, BMI, smoking status, and NSCLC staging were recorded. Of the 21 participants who went on to develop postoperative pulmonary complications, the percentage of predicted FEV1 was 88.8% compared with 87.5% in patients who did not develop complications. Predicted %FEV1 was not found to be a significant independent risk factor in determining PPC such as pneumonia, atelectasis, longer hospital stays in high-dependency units or admission to intensive care following VATS [3] (Table 1). Another study looked at 193 patients who underwent surgery for lung cancer. The rate of PPC in patients with an FEV1 <1.5L and ppoFEV1 <1.0L was 12.1% and 12.5% respectively and did not show a significant difference compared to patients without complications. Twenty-nine patients with PPC had a mean ppoFEV1 of 1.59 compared with 1.68 in 164 patients without complications. Using ppoFEV1 as part of preoperative risk assessments was not significant in predicting postoperative complications [4]. However, it should be highlighted that both these studies have small patient cohorts (n=285, with 21 patients experiencing PPC and n=193, with 29 patients experiencing PPC respectively) and were limitedly powered to detect a difference. 

Conversely, a retrospective study enrolling 956 patients investigated the risk factors for postoperative complications following pulmonary resection for NSCLC in Japan. Information on pulmonary function parameters such as percentage vital capacity (%VC) and %FEV1 was collected. A cut-off value of 70% for FEV1 was defined as obstructive ventilatory impairment. In Japan, FEV1 of less than 70% is defined as obstructive ventilatory impairment which is in contrast to the Global Initiative for Chronic Obstructive Lung Disease (GOLD) classification. Postoperative complications were present in 35% of patients with a %FEV1 <70% compared to 23% of patients with an FEV1 ≥70%. Univariate analysis showed that FEV1 <70% was associated with a greater risk of postoperative atelectasis, pneumonia, and air leakage. These findings oppose the aforementioned studies likely due to the larger population size and demonstrate %FEV1 as an important predictor and significant risk factor of postoperative complications [5].

Moreover, a study using whole lung CT to determine preoperative pulmonary function was carried out in 390 patients who underwent resection for lung cancer. The development cohort had 290 patients and 100 patients formed the validation cohort. Whole lung CT along with patient demographics was used to develop a regression equation for FEV1 to determine preoperative pulmonary function and ppoFEV1. The ppoFEV1 was also calculated based on the anatomic equation: measured %FEV1 x (42 - number of subsegments resected)/42. Both regression analysis and actual measured FEV1/FVC, %FEV1 and %ppoFEV1 were shown to be significant variables associated with predicting postoperative cardiopulmonary complications in both cohorts. Both calculated ppoFEV1 and actual ppoFEV1 were positively correlated with the occurrence of PPC [6]. The use of regression equations based on CT-derived parameters may be useful in preoperative risk assessment when spirometry is not feasible. 

Taking these studies into account, pulmonary function parameters continue to provide conflicting evidence for their ability to predict postoperative complications. The smaller population size of these studies is a limiting factor and we need stronger studies collaborated at national or international units to improve reliability

Diffusion capacity for carbon monoxide (DLCO)

In addition to spirometric parameters, the diffusion capacity for carbon monoxide (DLCO), also known as the transfer factor for carbon monoxide (TLCO), is considered in most preoperative assessments in addition to ppoFEV1. DLCO is based on the ability of carbon monoxide to bind to haemoglobin, therefore DLCO represents the efficiency of gas transfer from inspired air into red blood cells. A low DLCO predicts (a) a higher likelihood of pulmonary complications requiring either supplemental oxygen or hospitalization and (b) a worse dyspnea score. DLCO is also strongly associated with the risk of higher operative mortality [7]. DLCO is an independent predictor of postoperative morbidity despite normal spirometry as may be seen in patients with pulmonary fibrosis [8]. Thus, DLCO is important in predicting postoperative complications, including morbidity and mortality. Together with ppoFEV1, an estimated postoperative TLCO >40% is recognized as an average risk whereas a TLCO <40% is considered a high risk of developing PPC [2]. 

A prospective study involving 351 patients with NSCLC was conducted to determine the risk factors associated with PPC after lung cancer surgery. The preoperative evaluation demonstrated a lower predicted DLCO in the COPD group compared to non-COPD counterparts. Univariate analysis found that patients with COPD who went on to develop a PPC had a lower percentage of predicted DLCO of 86% compared to 93.1% in patients who did not develop PPC. Moreover, in the non-COPD group, predicted DLCO was 87.7% in patients who developed a PPC compared with 96.7% in patients who did not develop a PPC. This study signifies low DLCO as a vital risk factor for postoperative complications in both COPD and non-COPD groups thus highlighting the importance of measuring DLCO in patients with normal spirometry prior to resection for NSCLC [9]. 

DLCO was also found to be a significant prognostic factor following surgical resection for stage I NSCLC. In a study, 426 patients who underwent resection for stage I NSCLC were recruited to investigate the short and long-term outcomes in those with a 'marginal-risk' versus 'normal-risk'. Marginal-risk was defined as a ppoFEV1 of 30-60% and/or ppoDLCO of 30-60%; while, normal-risk was defined as ppoFEV1 ≥ 60% and ppoDLCO ≥ 60%. After applying exclusion criteria, 391 patients were included with 73 classified as marginal-risk and 318 as normal-risk. Overall, marginal-risk patients compared to normal-risk had higher postoperative morbidity (48% vs 21% respectively), pulmonary complications (34% vs 11%) and prolonged hospital stay (23% vs 6%). Among six patients with a ppoFEV1 and/or ppoDLCO of <40%, postoperative morbidity was found to be 83%. Furthermore, in the 27 patients with a ppoFEV1 and/or ppoDLCO of <50%, postoperative morbidity occurred in 44%, PPC occurred in 33% and 22% had longer hospital stays [10]. 

**Table 1:** Findings of the studies reviewed looking at the association between predicted FEV1 and DLCO and the rate of postoperative pulmonary complications. PPC: postoperative pulmonary complications; FEV1: forced expiratory volume in 1 second; ppoFEV1: predicted postoperative FEV1 (ppoFEV1); COPD: chronic obstructive pulmonary disease; DLCO: diffusion capacity for carbon monoxide

Study	No. of patients	Key findings
Agostini et al. 2018 [3]	285	Rate of PPC in patients with a mean predicted FEV1 of 88.8% was 7% (n=21)
Kaneda et al. 2020 [4]	193	Rate of PPC in patients with ppoFEV1 <1.5L was 17% (n=33)
		Rate of PPC in patients with ppoFEV1 <1.0L was 4% (n=8)
Motono et al. 2021 [5]	956	Rate of PPC in patients with FEV1 <70% was 35% (n=115)
		Rate of PPC in patients with FEV1 ≥70% was 23% (n=142)
Ueda et al. 2018 [6]	390	PPC occurred in 20% (n=20) of patients in the validation cohort with a ppoFEV1 of 77.2%
Kim et al. 2016 [9]	351	COPD patients with PPC (n=34) had a significantly lower predicted DLCO of 86% compared to non-PPC patients
		Non-COPD patients with PPC (n=23) had a significantly lower predicted DLCO of 87.7% compared to non-PPC patients
Ozeki et al. 2017 [10]	426	Rate of PPC was 33% (n=19) in patients with ppoDLCO <50%
		Rate of PPC was 83% (n=5) in patients with ppo-DLCO <40%

Cardiopulmonary exercise testing (CPET) 

Cardiopulmonary exercise testing (CPET) is a complex, non-invasive approach to cardiorespiratory evaluation during exercise and at rest under controlled physiological conditions [11]. Of late, CPET has been used for physiological assessment prior to thoracic surgery. CPET is a nonpareil entity for functional evaluation and risk stratification of patients prior to lung resections [12]. However, a major impediment is the restricted number of patients who are fit enough to undertake the test. It is based on the principle that system failure occurs when under stress. Monitoring of cardiopulmonary variables such as peak oxygen consumption (VO2), anaerobic threshold (AT), ventilatory equivalent for carbon dioxide (VE/VCO2 slope) can aid assessment [13, 14]. VO2 peak and AT are measures of a patient's exercise capacity whereas VCO2 demonstrates the gas exchange efficiency [14]. Theoretically, this can help predict post-thoracotomy morbidity and mortality as both thoracotomy and the immediate postoperative period represent demanding stress for both the circulatory and respiratory reserve [12].

VO2 peak is the maximum value of oxygen uptake measured during CPET at the end of the exercise phase [14]. It is the most used parameter when describing the capacity of surgery candidates. It is associated with postoperative mortality and morbidity, with separate predictive values for major surgical procedures [13]. One of the known disadvantages of this parameter is that it can be influenced by the subject's motivation, therefore causing an incorrect exclusion from a potentially curative procedure [13]. In addition, CPET is difficult to offer to patients unfit for the test, for example, patients with severe arthritis. Over the years controversies exist over the best cut-off which separates the lower risk from the intermediate/high risk for postoperative complications. Nonetheless in clinical practice, patients with VO2 max values > 20ml/kg/min (or 75% predicted) undergo major surgeries such as pneumonectomy whereas patients with values < 10ml/kg/min (or 35% predicted) seem to have better prognosis with oncological or palliative treatments [13]. 

Anaerobic/ lactate threshold (AT) is a measure of submaximal exercise capacity, i.e. oxygen consumption achieved almost exclusively under aerobic conditions [13]. Since it reflects the lactic acid serum level with metabolic acidosis under anaerobic situations, it is an excellent predictor of postoperative morbidity and mortality for significant elective procedures such as intra-abdominal surgical procedures and cardiovascular pathologies (such as myocardial ischemia and heart failure) [14]. Some sources from the literature have proposed that an AT < 11ml/kg/min is considered a prognostic marker of high surgical complications [13].

VE/VCO2 is not only a ratio of the minute ventilation to CO2 output but also an expression of gas exchange efficiency [13]. Elevated values of ventilatory equivalents were seen in several serious diseases such as heart failure, pulmonary hypertension and pulmonary fibrosis which can ultimately indicate a poor prognosis [13]. VE/VCO2 values used for triaging patients are different between studies, which ranged from 34-40 [13]. However, recent studies have shown that VE/VCO2 could be a more powerful indicator of short and long-term survival [13].

Cardiac and thoracic risk indices

As cardiovascular complications are potential risks following lung cancer resection, a cardiovascular risk assessment must be performed preoperatively [15]. The National Institute for Health and Care Excellence (NICE) set out several guidelines for assessing cardiovascular function prior to surgery. This includes waiting 30 days after a myocardial infarction to operate, seeking further review if a patient has 3 or more risk factors or poor cardiac function, and operating without further review if a patient has 2 or fewer risk factors and good cardiac function [16].

Thoracic Revised Cardiac Risk Index

The Revised Cardiac Risk Index (RCRI) was proposed in 1999 by Lee et al. for evaluation of stable non-urgent major non-cardiac surgery patients [17]. This was later revised in 2010 by Brunelli et al. into the Thoracic RCRI (ThRCRI), so it could be specifically used for a lung resection population [18]. The four factors that it entails are (1) history of ischemic heart disease (1.5 points) (2) history of cerebrovascular disease (1.5 points); (3) serum creatinine > 2mg/dL (1 point); (4) pneumonectomy (1.5 points).

The sum of these scores then divides patients into different risk classes that estimate their risk of cardiac complications, thus determining those who require further cardiac assessments: (1) Class A - 0 points (1.5%), (2) Class B - 1-1.5 points (5.8%), (3) Class C - 2-2.5 points (19%) (4) Class D - >2.5 points (23%).

The validity of the ThRCRI in predicting cardiovascular risk after major lung resection has been supported in multiple studies [19-20] (Table 2). This includes a retrospective study in which 4265 patients who underwent lung resection were analyzed from the American College of Surgeons National Surgical Quality Improvement Program (ACS NSQIP) database between 2005 and 2012. Patients were stratified into one of the four different classes (A-D) of the ThRCRI depending on their total risk factor score between 0-5.5 points. Cardiac complications in this study were defined as a myocardial infarction, cardiac arrest requiring cardiopulmonary resuscitation and pulseless ventricular tachycardia or ventricular fibrillation. Patients in risk class C and D had a higher rate of cardiac complications compared to A and B (9.1% & 3.6% vs 1.4% and 2.7%) [21].

**Table 2:** Percentage of patients with cardiac complications in each class of the ThRCRI score ThRCRI: Thoracic Revised Cardiac Risk Index

Reference	No. of Patients	ThRCRI Score (% of patients)	Outcome - % of patients with cardiac complications
Ferguson et al. 2014 [19]	26085	Class A (0) – 62.17%	Class A – 2.87%
		Class B (1-1.5) – 31.49%	Class B – 5.77%
		Class C (2.5) – 1.00%	Class C – 11.88%
		Class D (>2.5) – 5.34%	Class D – 11.12%
Brunelli et al. 2015 [20]	1370	Class A (0-1) – 77.52%	Class A – 4%
		Class B (1.5-2.5) – 20.73%	Class B – 11%
		Class C (>2.5) – 1.75%	Class C – 17%
Thomas et al. 2017 [21]	4265	Class A (0) – 78.00%	Class A – 1%
		Class B (1-1.5) – 19.00%	Class B – 3%
		Class C (2-2.5) – 0.50%	Class C – 9%
		Class D (>2.5) – 1.8%	Class D – 4%

Thoracic Surgery Risk Models

Many multivariable prediction models for mortality following lung resection have been produced. This includes Thoracoscore (The Thoracic Surgery Scoring System), which specifically looks at in-hospital mortality following general thoracic surgery. The nine variables it uses include age (<55, 55-65, >65), sex, American Society of Anesthesiologists (ASA) score (≤2, ≥3), Zubrod score (≤2, ≥3), dyspnea score (≤2, ≥3), the priority of surgery (elective, urgent/emergency) and procedure class (pneumonectomy, other) [22]. NICE recommends using models such as Thoracoscore to estimate mortality following lung resection for non-small-cell lung cancer [16].

Thoracoscore and the modified Thoracoscore (dyspnea score excluded) have previously been externally validated, including a retrospective study in which 1675 patients who underwent thoracic surgery (n=626 for lung resections) were analyzed from 2002 to 2006. The modified Thoracoscore proved to be an effective predictor of in-hospital mortality and midterm mortality (mean follow-up of 25 months), with in-hospital mortality risk increasing by 20% for every 1% increase of the modified Thoracoscore, and midterm mortality increasing by 12% for every 1% increase [23] (Table 3).

**Table 3:** Observed to expected ratio of mortality when using the modified Thoracoscore

Reference	No. of Patients	Observed:Expected Ratio of Mortality
Chamogeorgakis et al. 2007 [23]	1675	Low Risk - 0
		Medium Risk – 0.97
		High Risk – 0.99
Taylor et al. 2020 [24]	6600	Low Risk – 3.00
		Medium Risk – 1.29
		High Risk – 0.38

However, studies in recent years have shown the inaccuracy of Thoracoscore, particularly when compared to other models. One such study aimed to externally validate Thoracoscore, as well as other multivariable prediction models (modified Thoracoscore, Eurolung, modified Eurolung, European Society Objective Score and the Brunelli model), looking at their effectiveness in estimating perioperative mortality. The survival time (number of days from surgery to date of death) was measured in 6600 patients who underwent lung resection between 2012 and 2018. Each model was produced to predict either in-hospital or perioperative mortality; 5/6 of the models had an inaccurate estimation of the type of mortality they were made for, with the modified Eurolung model producing the only acceptable prediction (observed:expected ratio of 0.92). However, this model has limited variables and does not factor in patient comorbidities, thereby bringing its clinical validity into question [24].

A brief summary of some of the available risk models in thoracic surgery along with their disadvantages is mentioned in the table below [25-28] (Table 4). Their use, therefore, should not replace a more holistic approach, as stated by the model’s original authors [29].

**Table 4:** Summary of different available thoracic surgery risk models ASA: American Society of Anaesthesiologists; BMI: body mass index; CAD: coronary artery disease; CVD: cerebrovascular disease; ppoDLCO: predicted postoperative diffusion capacity of the lung for carbon monoxide; ppoFEV1: predicted postoperative forced expiratory volume in 1 second; PVD: peripheral vascular disease; VATS: video-assisted thoracoscopic surgery

Risk Models	Year published	Patient population	Database	Risk predictors	Outcome measured	Number or % of outcome	c-index	Disadvantages
Thoracoscore [22]	2007	15,183	Epithor database (French Society of Thoracic Surgery)	Age, sex, dyspnoea score, ASA score, performance status (PS), priority of surgery, diagnosis group, procedure class, and comorbid disease.	Inpatient mortality	338 (2.2%)	0.85	Only measures in-hospital mortality
European Society Objective Score (ESOS.01) [25]	2005	1694	European Society of Thoracic Surgeons (ESTS) database	Age and ppoFEV1	Inpatient mortality.	33 (1.9%)	0.739	Only measures in-hospital mortality
Society of Thoracic Surgery [26]	2016	27844	STS General Thoracic Surgery Database	Age, male gender, FEV1, body mass index, cerebrovascular disease, steroids, CAD, PVD, renal dysfunction, Zubrod score, ASA score, thoracotomy approach, induction therapy, reoperation, tumor stage, and greater extent of resection	Operative mortality, major morbidity	401 (1.4%); 2545 (9.1%)	0.78	Limited to 30-day follow-up
Eurolung2 [27]	2017	47960	ESTS database	Age, ppoFEV1, BMI, ASA, ppoDLCO, Male sex, CAD, CVD, pneumonectomy, thoracotomy approach and extended resections	30-day mortality, cardiopulmonary morbidity	1295 (2.7%); 8805 (18.4%)	0.74	Only measures in-hospital; limited to 30-day follow-up.
Brunelli model [28]	2020	732	institutionally maintained	Age >75, DLCO <70, ASA >2, PS >1, male sex and BMI <18.5	2-year survival	Score 1-3: 84%; Score >3: 66%	Not available	Needs external validation; single centre study; VATS lobectomies only

Frailty 

Frailty assessment is defined as an age-related decline in multiple physiological systems and can be used to flag vulnerable patients who may benefit from pre-operative rehab or non-surgical therapies [30]. The current frailty testing is divided into three categories -standard pre-operative testing, in-clinic, and prehospital admission assessments [31]. Examples of in-clinic assessments include The Modified Frailty Index (mFI), Revised Cardiac Risk Index (RCRI) and Charlson Comorbidity Index (CCI). Some prehospital admission tests are the six-minute walk test (6MWT), gait speed and hand-grip test [31].

mFI was formed by matching the Canadian Study of Health and Aging Frailty index (CSHA-FI) to the 11 variables, for example, MI history and diabetes, compiled by the American College of Surgeons NSQIP [31]. RCRI uses six independent components of major heart complications with each risk factor assigned a point. Patients with 0,1,2 or more risk factors are assigned respectively to classes I-IV [31]. CCI is a weighted index that predicts the cumulative mortality and 10-year survival of patients by considering the number and severity of comorbidities. 6MWT assessed the total distance walked which can vary from 380-782 m [31]. Gait speed on the other hand will be measured by an experienced physiotherapist in an empty hallway with 0 and 5m markings. The subject will be asked to walk down three times, allowing approximately 15s between trials, whilst the average gait speed is calculated for analysis [31]. A gait speed of <1m/s has been shown to identify high-risk participants. Grip strength was measured on the dominant hand with a hand-grip dynamometer, with the average calculated after three attempts [31].

A study on frailty assessment was conducted at a single-site thoracic surgical clinic - out of the 180 eligible patients, 125 completed screening. Results showed that 71 (57%) were prefrail and 15 (12%) were frail [30]. This is an interesting finding as in the study, a referral diagnosis of lung cancer and nodules is amongst the first and second most common: 46 (36.8%) for lung nodules and 40 (32%) for lung cancer [30]. Studies have shown that not only is frailty a relatively significant surgical risk factor common in a thoracic surgical clinic but it is also a feasible screening test as most patients were able to complete the assessment [30].

## Conclusions

Preoperative risk assessments for lung cancer resection play a vital role in determining whether it is appropriate to operate, deciding if further assessments or treatments are required, and predicting a patient’s prognosis. Some of these assessments, such as a frailty assessment, CPET and ThRCRI, have been externally validated by studies, proving their accuracy in predicting complications. Their use in combination, depending on the patient, would provide a detailed picture of what a patient’s outcome following surgery would be. However, other assessments, such as pulmonary function tests and Thoracoscore, still have conflicting evidence regarding their efficacy. This is possibly skewed by small underpowered studies, hence need for larger studies using acceptable cut off values. These assessments require further external validation, and should not, at this time, replace the use of the assessments that have proven to be effective.
